# The Impact of Biliary Drainage on Quality of Life in Unresectable Biliary and Pancreatic Cancer

**DOI:** 10.7759/cureus.78986

**Published:** 2025-02-14

**Authors:** Mohmad Sejarali Sayeed, Gnanadesigan Ekambaram, Jay Prakash S Rajput, Pooja Parmar, Swapnil Saikhedkar, Sajidali S Saiyad, Tehsin Saiyad

**Affiliations:** 1 Department of Gastrointestinal Surgery, Aryavart Hospital, Meerut, IND; 2 Department of Physiology, Nootan Medical College and Research Centre, Sankalchand Patel University, Visnagar, IND; 3 Department of Physiology, Pacific Medical College and Hospital, Udaipur, IND; 4 Department of Anatomy, J and D Institute of Nursing, Surat, IND; 5 Department of Microbiology, Veer Narmad University, Surat, IND

**Keywords:** biliary stenting outcomes, endoscopic retrograde cholangiopancreatography(ercp), fact-g: functional assessment of cancer therapy – general, fact-hs: functional assessment of cancer therapy - hepatobiliary symptom index, karnofsky performance status, malignant obstructive jaundice, palliative care in biliary malignancies, pancreatic cancer, percutaneous transhepatic biliary drainage(ptbd), visual analog scale for pruritus (vasp)

## Abstract

Introduction

Obstructive jaundice, a frequent complication of biliary tract and pancreatic malignancies, severely impacts patients’ quality of life (QoL). This condition is prevalent in unresectable cancers, often causing symptoms such as pruritus, anorexia, nausea, vomiting, and cholangitis, which hinder daily activities. Given the global burden of these cancers, the primary aim of the study was to evaluate the impact of biliary drainage on QoL and pruritus in patients with unresectable biliary and pancreatic malignancies. Secondary objectives include assessing changes in individual QoL domains and identifying factors associated with QoL outcomes.

Materials and methods

This prospective observational study was conducted at Meenakshi Mission Hospital and Research Centre, Madurai, from July 2020 to November 2021. The study included 82 patients with inoperable malignant biliary obstruction presenting with obstructive jaundice. Participants underwent either endoscopic retrograde cholangiopancreatography (ERCP; n=47, 57.3%) or percutaneous transhepatic biliary drainage (PTBD; n=35, 42.7%), depending on clinical suitability. QoL and pruritus were assessed at baseline, 1 week, and 4 weeks using validated tools (Functional assessment of cancer therapy-General (FACT-G), Functional assessment of cancer therapy-Hepatobiliary subscale (FACT-HS), and visual analog scale for pruritus (VASP)). Statistical analysis was performed using SPSS v26.0, with significance set at p < 0.05.

Results and discussion

The study involved 82 patients diagnosed with inoperable malignant biliary obstruction and presenting with obstructive jaundice. The mean age of the participants was 63.7 ± 10.5 years, with 58 (70.7%) of them being male. Type 2 diabetes (63.4%) and hypertension (52.4%) were prevalent comorbidities. Most patients had moderate functional impairment (64.6%).

Biliary drainage led to a significant reduction in bilirubin levels, decreasing from 13 mg/dL to 1.3 mg/dL by the fourth week (p < 0.001) but reducing social well-being (p < 0.001). FACT-G, FACT-HS, and hepatobiliary scores declined despite pruritus relief (p = 0.001). While emotional well-being (EWB) improved initially, its effect was not sustained at 4 weeks (p = 0.084), indicating persistent psychological distress.  Despite symptomatic relief, overall QoL declined, emphasizing the need for a multidisciplinary approach incorporating palliative care, nutritional support, and psychological counseling to improve long-term patient outcomes.

Conclusion

The study demonstrates that while biliary drainage significantly improves physical well-being, as evidenced by the reduction in bilirubin levels, there is a decline in the other aspects of QoL, highlighting the complexity of symptom management in patients with malignant biliary obstruction. These findings highlight the need for a comprehensive approach that addresses multiple aspects of well-being in this patient population.

## Introduction

Obstructive jaundice is a debilitating complication of biliary tract and pancreatic malignancies, significantly impairing quality of life (QoL). According to the Global Burden of Diseases (GBD) study, cancer is the second leading cause of mortality worldwide, with biliary tract and pancreatic cancers contributing substantially to this burden [1]. In patients with unresectable malignancies, post-biliary drainage QoL assessment is crucial, as treatment goals extend beyond survival to symptom relief and overall well-being.

Obstructive jaundice is the primary complaint in over 90% of these patients [2], causing pruritus, fever, anorexia, nausea, vomiting, and cholangitis, all of which severely limit daily activities. In India, gallbladder cancer is particularly prevalent in northern and northeastern regions and is strongly linked to gallstone disease and chronic infections with *Helicobacter pylori* and *Salmonella typhi* [3,4]. While pancreatic cancer is less common, its prognosis remains poor, with a five-year survival rate of only 5% [4,5]. The presence of obstructive jaundice worsens hepatic dysfunction and systemic complications, further diminishing QoL [6].

Palliative biliary drainage procedures, including endoscopic stent placement (ESP) and percutaneous transhepatic biliary drainage (PTBD), are commonly used to relieve obstruction and improve symptoms. While ESP is minimally invasive, it carries risks such as stent migration, pancreatitis, perforation, and infections. PTBD serves as an alternative when ESP is ineffective and is particularly effective in alleviating jaundice and improving liver function in patients with malignant biliary obstruction. However, recent studies highlight the need to consider its broader psychosocial impact in palliative care. Some research suggests QoL improvement post-drainage, though outcomes vary. These variations arise due to procedural factors - ESP is preferred for distal obstructions with a shorter recovery time, though higher reintervention rates may reduce long-term QoL benefits. PTBD, often necessary for proximal tumors or failed ESP cases, can be limited by external drainage discomfort and infection risks. Additionally, patient-specific factors such as tumor location, functional status, and infection risk significantly influence outcomes. A study by Robson et al. (2010) found that despite improvements in pruritus, overall QoL scores declined following PTBD, underscoring the complex nature of well-being in patients with advanced malignancy [7].

Several validated scales, including Karnofsky Performance Status (KPS), Functional Assessment of Cancer Therapy-General (FACT-G), Functional Assessment of Cancer Therapy-Hepatobiliary (FACT-Hep), and visual analog scale for pruritus (VASP), assess physical, psychological, and social health. However, QoL studies in patients with unresectable biliary and pancreatic malignancies remain limited. This study aims to assess the impact of biliary drainage on QoL, evaluate symptomatic relief post-drainage, and understand the psychological and functional changes post-treatment. The objectives of the study are to compare QoL before and after biliary drainage and quantify the reduction in serum bilirubin levels.

## Materials and methods

Study design and setting

This prospective observational study was conducted in the Department of Surgical Gastroenterology at Meenakshi Mission Hospital and Research Centre, Madurai, between October 2020 and November 2021. Ethical approval was obtained from the Institutional Ethics Committee of Meenakshi Mission Hospital and Research Centre, Madurai, Tamil Nadu, on 28-09-2020. The study aimed to evaluate the impact of biliary drainage on QoL and pruritus in patients with malignant biliary obstruction.

Study population

The study included both inpatients and outpatients with inoperable malignant biliary obstruction presenting with obstructive jaundice. Patients eligible for surgical resection were excluded. Informed written consent was obtained from all participants before enrollment.

Sample size calculation

The required sample size was determined to detect a clinically significant change in QoL, assessed using the FACT-G questionnaire. Based on previous studies [7,8,9], the minimally important difference (MID) for FACT-G was set at 7 points, with an expected standard deviation of 22.8.

Assuming a power of 80% (β = 0.20) and a significance level of 5% (α = 0.05), the sample size was calculated using the formula for comparing two means: 

\( n = \left(\frac{Z_{\alpha/2} + Z_{\beta}}{\Delta / \sigma}\right)^2 \)

where: 

Zα/2=1.96 (for a two-sided test at α = 0.05), 

Zβ=0.84(for 80% power), 

Δ=7 (MID for FACT-G), 

σ=22.8(standard deviation of the FACT-G score). 

Hence, 

\( n = \left(\frac{1.96 + 0.84}{7 / 22.8}\right)^2 \)

The minimum required sample size was 62 participants per group. To account for a 20% attrition rate, the final sample size was adjusted to approximately 78 participants. Ultimately, 82 participants were included to ensure robust results, aligning with recommendations for studies involving patients with advanced malignancies [7,9]. The initial sample size calculation was based on the FACT-G MID, standard deviation, and statistical power considerations. To maintain statistical validity despite potential patient loss, an estimated 20% dropout rate was incorporated into the calculation. However, no dropouts occurred during the study period, likely due to the short follow-up duration of four weeks, which was deliberately chosen to minimize attrition rather than including a long-term follow-up. Consequently, all enrolled patients completed the follow-up assessments, strengthening the reliability of the findings.

Procedures

Participants underwent either endoscopic or percutaneous biliary drainage, depending on their clinical condition. QoL and pruritus were assessed pre-procedure, at 1 week, and at 4 weeks post-procedure using validated tools: the Functional Assessment of Cancer Therapy-Hepatobiliary subscale (FACT-HS) questionnaire and the VASP. Clinical follow-up was conducted through patient interviews and chart reviews. Procedural and post-procedure complications were documented. This study followed patients for a maximum of 4 weeks post-procedure, focusing on clinical and QoL outcomes. Long-term survival and mortality data were not collected.

Data collection

Patients underwent either endoscopic or percutaneous biliary drainage based on their individual condition. After obtaining informed consent, participants completed the questionnaires mentioned below.

As this was a prospective observational study, no randomization was performed. However, to minimize selection bias, we implemented strict inclusion criteria and ensured that the sample reflected the typical population of patients with malignant biliary obstruction.

The KPS was used to assess functional impairment and the ability to carry out daily activities in patients with unresectable biliary and pancreatic cancer. KPS was assessed at baseline to establish the initial functional status of patients before biliary drainage. The scale ranges from 0 (death) to 100 (normal functioning), with scores classified as mild (80-100), moderate (50-70), and severe impairment (<50). It is widely used in oncology and palliative care to evaluate disease burden and treatment efficacy.

Functional Assessment of Cancer Therapy-Hepatobiliary (FACT-HS): A 45-item self-report instrument measuring QoL in hepatobiliary cancer patients. It includes FACT-General (FACT-G): 27 items assessing physical, social/family, functional, and emotional well-being (EWB), hepatobiliary subscale (HS): Disease-specific questions on pruritus, jaundice, and drainage catheters, VASP assessment: A 4-item scale measuring itch sensation on a 10-point scale (0 = no itch, 10 = very strong itch).

Clinical follow-up was conducted at baseline, one week, and four weeks post-procedure through structured patient interviews and medical chart reviews. Patient interviews utilized validated tools (FACT-G, FACT-HS, and VASP) to assess physical symptoms, EWB, and overall QoL. Chart reviews recorded bilirubin levels, procedural outcomes, complications, and medication adjustments, ensuring objective correlation with patient-reported data.

Statistical analysis

The Kolmogorov-Smirnov (K-S) test was used to assess the normality of continuous variables, as it helps determine whether the data follow a normal distribution, which is essential for choosing the appropriate statistical tests. Since the QoL and VASP scores were non-normally distributed, they were summarized using medians and interquartile ranges (IQR) instead of means and standard deviations. The Friedman test, a non-parametric equivalent of repeated-measures analysis of variance (ANOVA), was used to evaluate overall changes in bilirubin levels, QoL, and VASP scores over time, as it is suitable for comparing multiple related samples when normality assumptions are not met. For pairwise comparisons between time points (baseline vs. 1-week, baseline vs. 4-week), the Wilcoxon signed-rank test was applied because it accounts for paired, non-normally distributed data and measures changes within the same individuals. Non-parametric tests were used due to the non-normal distribution of the data. Although multiple pairwise comparisons were performed, no corrections for multiple comparisons (e.g., Bonferroni) were applied to avoid unnecessary complexity. This decision was made based on the exploratory nature of the study, where the focus was on identifying general trends rather than precise comparisons. We believe the risk of type I error is minimal in this context. Data analysis was performed using SPSS v26.0. Statistical significance was set at p < 0.05. Results were presented using appropriate pie charts and bar graphs.

## Results

Patient demographics and clinical characteristics

A total of 82 patients were included in the study, with a mean age of 63.7 ± 10.5 years. The study population comprised 58 males (70.7%) and 24 females (29.3%), with the mean age for males being 63.9 ± 10.7 years and for females 63.4 ± 11.0 years (Mean ± SD). An independent t-test revealed no statistically significant difference in age between males and females (t = 0.214, p = 0.831), confirming a similar age distribution across genders.

Table 1 illustrates the demographic distribution of the study population based on age and gender. The majority of patients belong to the 61-70 age group (42.7%), followed by the 51-60 age group (26.8%). The male-to-female ratio is approximately 2.4:1, with males comprising 70.7% and females 29.3% of the total sample. 

**Table 1:** Demographic Distribution of Patients by Age and Gender Data are presented as number (n) and percentage (%). Percentages represent the proportion of patients within each age group and gender category relative to the total study population (N = 82).

Age Group	No. of Patients, n (%)	Males, n (%)	Females, n (%)
31-40	1 (1.2%)	1 (100.0%)	0 (0.0%)
41-50	6 (7.3%)	4 (66.7%)	2 (33.3%)
51-60	22 (26.8%)	15 (68.2%)	7 (31.8%)
61-70	35 (42.7%)	25 (71.4%)	10 (28.6%)
71-80	13 (15.9%)	9 (69.2%)	4 (30.8%)
> 80	5 (6.1%)	4 (80.0%)	1 (20.0%)
Total	82 (100.0%)	58 (70.7%)	24 (29.3%)

Among the primary diagnoses, pancreatic cancer was the most common, affecting 29 (35.4%) patients, followed by cholangiocarcinoma in 21 (25.6%), ampullary/periampullary carcinoma in 20 (24.4%), gallbladder cancer in 6 (7.3%), and metastatic disease in 6 (7.3%). These findings align with the expected distribution of malignant biliary obstructions in clinical practice.

Comorbidities were prevalent, with type 2 diabetes mellitus reported in 52 (63.4%) patients and hypertension in 43 (52.4%). Other significant conditions included coronary artery disease in 23 (28.0%), hypothyroidism in 9 (11.0%), and chronic kidney disease in 4 (4.9%). Additionally, 24 (29.3%) patients had other chronic illnesses, underscoring the substantial comorbidity burden in this population.

During the four-week follow-up period, no deaths were recorded. Since the study primarily focused on short-term clinical outcomes, long-term survival data were not collected.

Functional status and interventions

The KPS scale indicated that most patients had moderate functional impairment, observed in 53 (64.6%). Severe impairment was seen in 16 (19.5%), while 13 (15.9%) had mild impairment. The mean KPS score was 60.9 ± 18, suggesting that the majority of patients experienced moderate limitations in daily activities due to advanced malignancies.

Regarding interventions, endoscopic retrograde cholangiopancreatography (ERCP) was performed in 47 (57.3%) patients, whereas PTBD was required for 35 (42.7%). ERCP was the preferred approach due to its minimally invasive nature, with PTBD used when ERCP was not feasible.

The primary indication for biliary drainage was jaundice with or without pruritus in 61 (74.4%) patients. Other indications included jaundice with pedal edema in six (7.3%), cholangitis with pruritus in five (6.1%), and pain with jaundice or fever in four (4.8%). Only one (1.2%) patient presented without specific symptoms.

In terms of biliary obstruction location, low-level obstruction was the most frequent, seen in 50 (61.0%) cases, while high-level and mid-level obstructions were identified in 20 (24.4%) and 12 (14.6%), respectively.

Biochemical response to biliary drainage

A significant reduction in bilirubin levels was observed following biliary drainage, confirming its efficacy in restoring bile flow. The median bilirubin levels decreased from 13 mg/dL (IQR: 10.4-17.8) at baseline to 6.1 mg/dL (IQR: 4.9-10) at one week and further to 1.3 mg/dL (IQR: 0.9-2.1) at four weeks (p < 0.001 for both reductions). These findings demonstrate substantial biochemical improvement post-intervention.

Quality of life (QoL) outcomes

**Figure 1:**
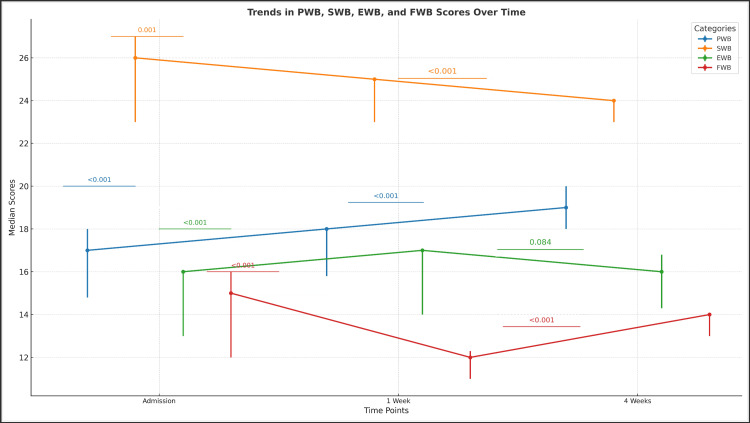
Trends in Physical, Social, Emotional, and Functional Well-Being Scores Over Time The data are represented as median (IQR). The statistical significance of changes over time (Admission, 1 Week, and 4 Weeks) was calculated using the Wilcoxon signed-rank test for paired non-parametric data. Test statistics (Z-values) are indicated alongside the p-values. Statistical significance was considered at p < 0.05 for all comparisons, with highly significant values denoted as p < 0.001. For clarity, error bars represent the IQR, and p-values between time points are shown for each category: PWB: Physical Well-Being; SWB: Social Well-Being; EWB: Emotional Well-Being; FWB: Functional Well-Being. The image was created by the authors of this article.

QoL was evaluated across multiple domains, including physical, social, emotional, and functional well-being. The findings, summarized in Table 2 and visualized in Figure 1, indicate notable improvements in some aspects of well-being following biliary drainage. Physical well-being showed progressive improvement, suggesting relief from symptoms over time. In contrast, social well-being declined gradually, reflecting increasing challenges in social support and interactions. EWB exhibited improvement at one week post-procedure; however, no significant changes were observed at four weeks, suggesting that early symptom relief was not sustained. Functional well-being initially declined, likely due to procedural recovery, but showed signs of improvement by the fourth week, indicating an adjustment period post-intervention.

**Table 2:** Quality of Life Metrics Over Time Data are represented as median (IQR). The p-values and test statistics were calculated using the Wilcoxon signed-rank test. Statistical significance was defined as p < 0.05. The Z-value is presented as the test statistic for comparisons between values at presentation and subsequent time points (1 week and 4 weeks). PWB: Physical Well-Being; SWB: Social Well-Being; EWB: Emotional Well-Being; FWB: Functional Well-Being

Quality of Life Domains	Time Point	Median	IQR	Test Statistic (Z-value)	Presentation * 1 Week (p)	Presentation * 4 Week (p)
PWB Scores	At Presentation	17	14.8–18.0	-4.23	<0.001	<0.001
	1 Week	18	15.8–18.0			
	4 Weeks	19	18.0–20.0			
SWB Scores	At Presentation	26	23.0–27.0	-3.29	0.001	<0.001
	1 Week	25	23.0–25.0			
	4 Weeks	24	23.0–24.0			
EWB Scores	At Presentation	16	13.0–16.0	-4.10	<0.001	0.084
	1 Week	17	14.0–17.0			
	4 Weeks	16	14.3–16.8			
FWB Scores	At Presentation	15	12.0–16.0	-4.35	<0.001	<0.001
	1 Week	12	11.0–12.3			
	4 Weeks	14	13.0–14.0			

Functional assessment of cancer therapy-General (FACT-G), hepatobiliary, functional assessment of cancer therapy-Hepatobiliary subscale (FACT-HS), and visual analog scale for pruritus (VASP) scores

**Figure 2:**
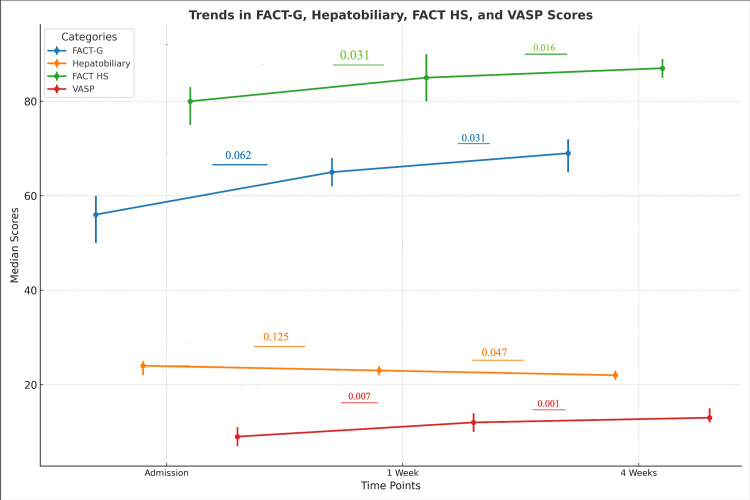
Trends in FACT-G, Hepatobiliary, FACT-HS, and VASP Scores Over Time Data are represented as median (IQR). The p-values and test statistics (Z-values) were calculated using the Wilcoxon signed-rank test, which is appropriate for paired non-parametric data. Statistical significance was set at p < 0.05, with highly significant results denoted as p < 0.001. The error bars indicate the IQR for each time point, and the p-values with corresponding Z-values highlight significant differences between scores at Admission, 1 Week, and 4 Weeks for the following quality-of-life measures: FACT-G: Functional Assessment of Cancer Therapy – General; FACT-HS: Functional Assessment of Cancer Therapy - hepatobiliary subscale, assessing symptoms specific to hepatobiliary cancers; VASP: Visual Analog Scale for Pruritus, assessing pruritus intensity. The image was created by the authors of this article.

Changes in FACT-G, Hepatobiliary, FACT-HS, and VASP scores were assessed at baseline, one week, and four weeks post-procedure, as detailed in Table 3 and illustrated in Figure 2. FACT-G scores demonstrated a gradual decline, with a significant decrease by four weeks, suggesting an overall reduction in QoL over time. The HS reflected a significant reduction in scores at four weeks, indicating worsening disease-specific symptoms. FACT-HS scores also showed a steady decline, with significant reductions observed at both one and four weeks. The most notable improvement was observed in pruritus relief, as reflected in the consistent reduction in VASP scores. This confirms the effectiveness of biliary drainage in alleviating pruritus-related distress, underscoring its role in symptomatic relief despite the progressive nature of the underlying disease.

**Table 3:** Changes in Quality-of-Life Scores Over Time for FACT-G, Hepatobiliary, FACT-HS, and VASP Tools Data are represented as median (IQR). The p-values and test statistics (Z-values) were calculated using the Wilcoxon signed-rank test, which is suitable for comparing paired non-parametric data. Statistical significance was set at p < 0.05, and the Z-value indicates the test statistic for comparisons between the time of presentation and 1-week or 4-week time points. FACT-G: Functional Assessment of Cancer Therapy-General, measuring general quality of life; FACT-HS: Functional Assessment of Cancer Therapy-Hepatobiliary Subscale, a detailed measure of symptom burden. VASP: Visual Analog Scale for Pruritus, measuring Pruritus.

Quality of Life Assessment Tools	Time Point	Median	IQR	Test Statistic (Z-value)	1-Week P-value	4-Week P-value
FACT-G	At Presentation	75	66–80	-1.85	0.062	0.031
	1 Week	70	65–78			
	4 Weeks	65	60–70			
Hepatobiliary Subscale	At Presentation	30	27–29	-1.54	0.125	0.047
	1 Week	28	25–27			
	4 Weeks	25	22–26			
FACT-HS	At Presentation	105	103–104	-2.16	0.031	0.016
	1 Week	100	97–99			
	4 Weeks	95	90–94			
VASP	At Presentation	7	6–7	-2.72	0.007	0.001
	1 Week	6	5–6			
	4 Weeks	5	4–5			

## Discussion

Demographic characteristics and gender disparities

The study cohort had a mean age of 63.7 years (SD = 10.5), aligning with the typical demographic affected by biliary and pancreatic malignancies. The majority, 35 (42.7%), were aged 51-70 years, consistent with findings from Barkay et al. [9], which identify the sixth and seventh decades as peak incidence periods for these conditions. The inclusion of seven (8.5%) younger patients (<50 years) and five (6.1%) elderly patients (>80 years) highlights the need for age-specific treatment approaches to optimize patient outcomes.

A male predominance was observed, with 58 (70.7%) males and 24 (29.3%) females, reflecting well-established epidemiological trends in biliary and pancreatic malignancies. This disparity, often attributed to genetic, hormonal, and lifestyle factors, such as smoking and alcohol use, has been reported by Barkay et al. [9] and Castiglione et al. [10]. These findings emphasize the importance of gender-specific prevention, diagnostic, and treatment planning strategies for these cancers.

Diagnostic distribution and clinical implications

The diagnostic distribution in this study aligns with global data on malignant biliary obstruction, with 29 (35.4%) patients diagnosed with pancreatic cancer, followed by 21 (25.6%) with cholangiocarcinoma and 20 (24.4%) with ampullary/periampullary cancers. Gallbladder cancer and metastatic disease were less common, each affecting six (7.3%) patients. These findings mirror those of Castiglione et al. [10] but differ from Gamanagatti et al. [11], who reported a higher prevalence of gallbladder cancer (71.4%), and Barkay et al. [9], who observed a 63.4% prevalence of pancreatic cancer-nearly double the rate observed in our cohort.

The high prevalence of pancreatic cancer aligns with studies such as Lillemoe et al. (1999) [12], which examined surgical palliation, including biliary drainage, in unresectable periampullary adenocarcinoma. These findings reinforce the complexity of managing malignant biliary obstructions and highlight the necessity of tailored treatment strategies to optimize patient outcomes.

Functional status and interventional approaches

The KPS distribution revealed significant functional impairment, with 53 (64.6%) of patients experiencing moderate impairment, 16 (19.5%) having severe impairment, and 13 (15.9%) classified as having mild impairment. The mean KPS score (60.9, SD = 18) suggests that the majority of patients required biliary drainage to alleviate symptoms and enhance QoL. These findings contrast with those of Robson et al. [7], who reported a higher baseline KPS of 74%, possibly due to differences in disease stage or patient selection criteria.

ERCP was the preferred procedure, performed in 47 (57.3%) of cases, reaffirming its minimally invasive nature and high success rate. These results align with Saluja et al. (2007) [13], who demonstrated superior QoL outcomes for endoscopic compared to percutaneous drainage in malignant biliary obstruction. Speer et al. (1987) [14] highlighted the importance of stent selection, particularly recommending a 10F gauge stent for optimal procedural success.

PTBD was performed in 35 (42.7%) of cases, often as a secondary approach following failed ERCP or for patients with complex anatomical obstructions. These findings are supported by van der Gaag et al. (2010) [15], who emphasized the importance of early drainage in pancreatic cancer patients to mitigate complications related to biliary obstruction.

Biliary drainage effectiveness

The effectiveness of biliary drainage was demonstrated through a significant reduction in bilirubin levels, which decreased from 13 mg/dL at baseline to 1.3 mg/dL (IQR: 0.9-2.1) at four weeks. This decline, consistent with previous studies such as Robson et al. [7], confirms the role of biliary decompression in symptom relief and highlights its palliative effectiveness in malignant obstruction.

QoL outcomes

QoL assessments revealed a statistically significant improvement in physical well-being (p < 0.001) over four weeks, likely due to symptom relief from jaundice and pruritus. In contrast, social well-being declined (p < 0.001), suggesting a reduction in social interactions due to disease progression or procedural limitations. EWB showed an early improvement at one week (p < 0.001) but was not sustained at four weeks (p = 0.084), emphasizing the need for continued psychological support. Functional well-being initially declined (p < 0.001) but improved at four weeks, reflecting an adjustment period post-intervention. These findings are consistent with previous studies, including Castiglione et al. [10], Gamanagatti et al. [11], and Abraham et al. [16], which reported similar QoL trends following biliary drainage procedures.

The FACT-G score showed a gradual decline, decreasing from 75 at baseline to 70 at one week (p = 0.062) and 65 at four weeks (p = 0.031). This trend aligns with findings from Robson et al. [7] and Barkay et al. [9], suggesting that biliary drainage alleviates physical symptoms but does not significantly enhance overall QoL in the short term due to advanced disease progression and procedural burdens.

The HS exhibited a similar trend, declining from 30 at baseline to 28 at one week (p = 0.125) and 25 at four weeks (p = 0.047), consistent with Robson et al. [7] and Salmanroghani et al. [17], who observed a high symptom burden in pancreatic cancer patients.

Pruritus relief, assessed by the VASP, showed significant improvement, with scores decreasing from 7 at baseline to 6 at one week (p = 0.007) and 5 at four weeks (p = 0.001). These findings reinforce the effectiveness of biliary decompression in alleviating pruritus-related distress, consistent with studies by Robson et al. [7], Barkay et al. [9], and Salmanroghani et al. [17]. Our QoL findings align with Dumonceau et al. [18], who provide guidelines on stenting indications and clinical outcomes. Additionally, Sato et al. [19] offer insights into the long-term effectiveness of biliary drainage based on follow-up data from endoscopic stenting for bile duct stones.

Biliary drainage effectively reduces bilirubin levels and alleviates pruritus, but overall QoL continues to decline, likely due to disease progression and procedural burden. These findings underscore the need for multidisciplinary palliative care strategies to improve long-term outcomes in patients with malignant biliary obstruction [20].

While biliary drainage effectively alleviates jaundice and improves liver function, our findings indicate a decline in social well-being following the procedure. This paradox may be attributed to several factors. Despite physical symptom relief, patients with advanced malignancy continue to experience fatigue, emotional distress, and reduced mobility, which can hinder social engagement. Additionally, prolonged hospital stays, frequent medical follow-ups, and increased dependence on caregivers may contribute to a sense of isolation and diminished social interaction. Prior studies have highlighted that patients undergoing palliative interventions often struggle with adjusting to new physical limitations and psychological burdens, which may explain the observed decline in social QoL. Moreover, the progressive nature of the underlying disease remains a key factor, as ongoing symptoms and uncertainty about prognosis may further impact patients' willingness or ability to participate in social activities. These findings emphasize the need for a holistic approach to care that integrates psychological support, social reintegration strategies, and tailored interventions to enhance overall well-being beyond physical symptom management.

Limitations and future directions

This study has several limitations that affect the generalizability and interpretation of findings. The small sample size and single-center design limit broader applicability, necessitating larger, multi-center studies. Additionally, the short follow-up period (4 weeks) captures only immediate post-procedural outcomes, restricting insights into long-term symptom relief, complications, functional stability, and survival outcomes. Limited logistics prevented long-term follow-up, but future studies should include survival data to distinguish the effects of disease progression from procedure-related outcomes.

The absence of a control group prevents direct comparisons with conservative management, making causal inferences difficult. Additionally, selection bias may be present, as ERCP and PTBD were assigned based on clinical suitability rather than randomization. Future randomized controlled trials (RCTs) or matched cohort studies could help mitigate this bias. The heterogeneous study population, including various biliary and pancreatic malignancies, may have influenced outcomes. Subgroup analyses could clarify differences by cancer type.

The study did not evaluate the impact of chemotherapy or radiation therapy on QoL, an important factor in multimodal cancer care. Furthermore, mortality data were not recorded, limiting survival analysis. Post-procedural complications were not systematically collected, though we acknowledge their potential influence on QoL. Future studies should incorporate complication data, blinded QoL assessments, and explore AI-driven predictive modeling for patient selection.

Despite these limitations, this study provides valuable insights into biliary drainage outcomes and highlights the need for a comprehensive, patient-centered approach incorporating multidisciplinary interventions such as psychological counseling and nutritional support to optimize patient care.

## Conclusions

Biliary drainage is a critical intervention for symptom management in patients with unresectable biliary and pancreatic cancer, particularly in alleviating pruritus and enhancing physical well-being. While its effectiveness in providing immediate symptom relief is well-documented, improvements in emotional, social, and functional well-being remain inconsistent. This underscores the need for complementary interventions, such as psychological counseling, nutritional support, and comprehensive palliative care, to address the broader impact of the disease.

Despite its role in reducing jaundice and pruritus, biliary drainage does not consistently lead to long-term QoL improvements. Declining FACT-G, FACT-HS, and hepatobiliary scores suggest that while temporary symptom relief is achieved, factors such as psychological distress, fatigue, and disease progression continue to affect well-being. A multidisciplinary, patient-centered approach that integrates symptom management, psychosocial support, and palliative care is essential for optimizing outcomes. Future research should focus on long-term, multi-center studies integrating randomized designs, survival data, and personalized treatment approaches to optimize patient outcomes.
